# Supplementary material for: Homeostatic membrane tension constrains cancer cell dissemination by counteracting BAR protein assembly
Source: Nat Commun. 2021 Oct 11;12:5930. doi: 10.1038/s41467-021-26156-4 (PMC8505629; doi:10.1038/s41467-021-26156-4)
Supplement: Supplementary file 1 — Supplementary Information [file 41467_2021_26156_MOESM1_ESM.pdf]

Supplementary information

Homeostatic membrane constrains cancer cell dissemination by counteracting BAR protein assembly

Kazuya Tsujita[1,2,3*], Reiko Satow[4], Shinobu Asada[4], Yoshikazu Nakamura[4,5], Luis Arnes[6], Keisuke Sako[7], Yasuyuki Fujita[8], Kiyoko Fukami[4], and Toshiki Itoh[1,2]

[1]Biosignal Research Center, Kobe University, Kobe, Hyogo, 657-8501, Japan.

[2]Department of Biochemistry and Molecular Biology, Kobe University Graduate School of Medicine, Kobe, Hyogo 650-0017, Japan.

[3]AMED-PRIME, Japan Agency for Medical Research and Development, Tokyo 100-0004, Japan.

[4]Laboratory of Genome and Biosignals, Tokyo University of Pharmacy and Life Sciences, Hachioji, Tokyo 192-0392, Japan.

[5]Department of Applied Biological Science, Faculty of Science and Technology, Tokyo University of Science, Noda, Chiba, 278-8510, Japan.

[6]The Novo Nordisk Foundation Center for Stem Cell Biology (DanStem), Biotech Research & Innovation Centre, University of Copenhagen, Copenhagen, Denmark

[7]National Cerebral and Cardiovascular Center Research Institute, Osaka 565-8565, Japan

[8] Division of Molecular Oncology, Graduate School of Medicine, Kyoto University, Kyoto 606-8501, Japan.

* Correspondence to Kazuya Tsujita: (e-mail: tsujita@people.kobe-u.ac.jp)

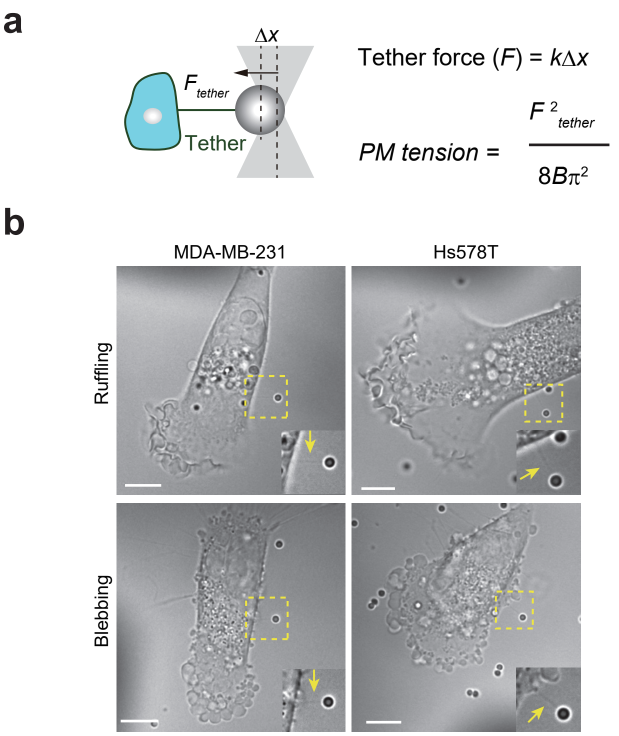

c

Cell type	Tether force (pN)	PM tension (pN/ µm)
MCF10A	31.76	91.89
MDCK II	31.2	90.42
IAR-2	33.1	102.6
AU565	27.92	73.09
MCF7	29.79	82.78
MDA-MB-231 (ruffling)	22.15	45.19
MDA-MB-231 (blebbing)	23.45	50.45
Hs578T (ruffling)	21.89	44.05
Hs578T (blebbing)	23.36	50.43
PC-3 (ruffling)	20.32	38.33
PC-3 (Blebbing)	21.44	42.61
PANC-1(ruffling)	21.88	45.08

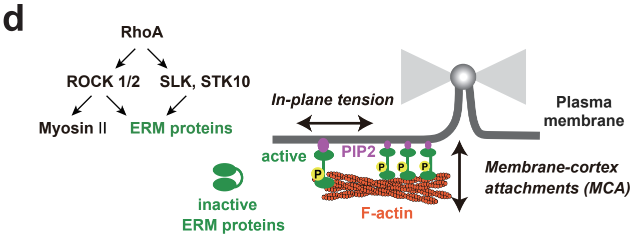

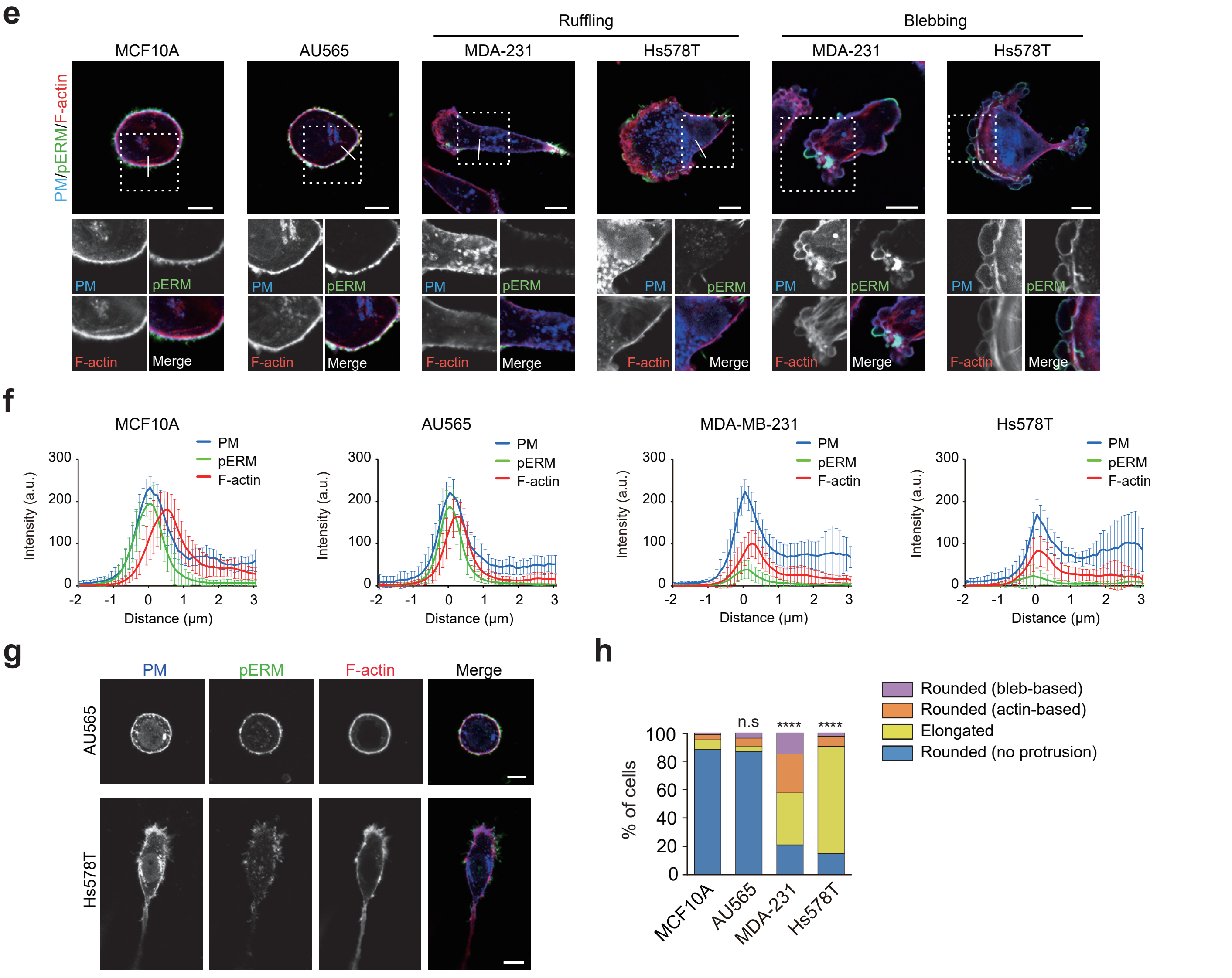

Tsujita et al, Supplementary Fig.1

Supplementary Fig. 1 Analysis of the tether force and PM tension

a Schematic illustration of the measurements of tether force (F_{tether}) with optical tweezers. k is the stiffness of the trap and Δx is the displacement of the bead from the trap center. PM tension can be estimated with the formula as described. B is the bending stiffness of the membrane. See the Methods section for details. **b** Time-lapse images of ruffling and blebbing cells in optical tweezers experiments. Yellow arrows indicate membrane tether. See Supplementary Movie 1. Images are representative of two independent experiments with similar results. Scale bars, 10 μm. **c** Mean values of tether force and PM tension in Fig. 1**a**. **d** Schematic illustration of PM tension regulation by membrane-to-cortex attachment (MCA) via ERM proteins. ERM proteins are activated by RHOA through ERM kinases, including ROCK1/2, SLK, and STK10. **e** Confocal images of indicated cells stained with anti-pERM antibodies, phalloidin, and wheat germ agglutinin (WGA). See also Fig. 1b. Scale bars, 10 μm. **f** Mean fluorescence intensity of the line scan across the PM on the lateral side, as indicated by the white lines in (**e**). n = 10 (MCF10A), n = 10 (AU565), n = 10 (MDA-MB-231), and n = 10 (HS578T) cells from three independent experiments. **g** Confocal images of AU565 or Hs578Tcells stained with anti-pERM antibodies, phalloidin, and WGA in a 3D collagen matrix. Scale bars, 10 μm. **h** Quantification of protrusions of the indicated cells in a 3D collagen matrix (3D). n = 144 (MCF10A), n = 107 (AU565), n = 175 (MDA-MB-231), and n = 126 (Hs578T) cells from two independent experiments. Chi-square test. n.s., not significant; ****$P < 0.0001$.

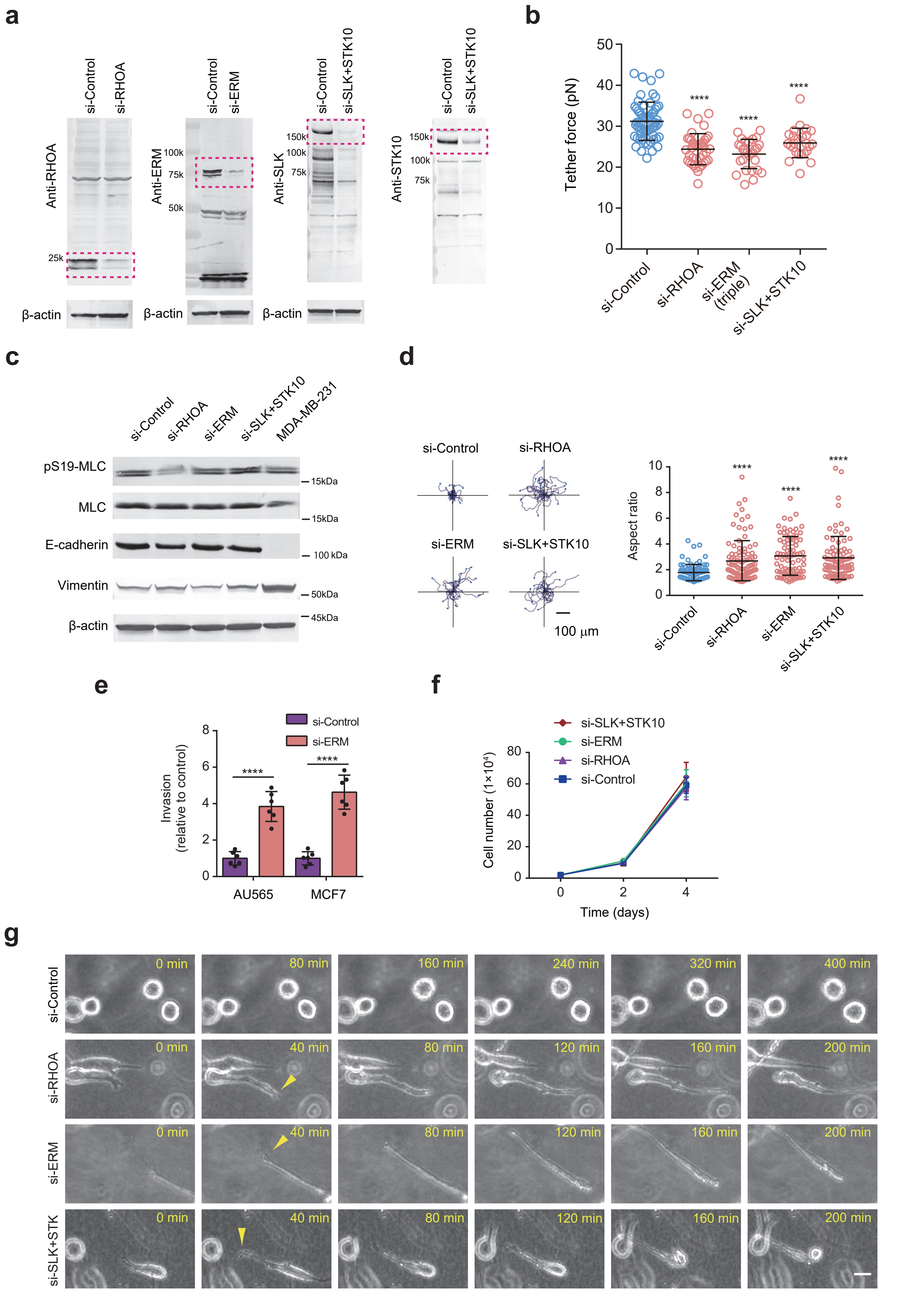

Tsujita et al, Supplementary Fig. 2

Supplementary Fig. 2 A decrease in PM tension induces a mesenchymal migratory phenotype in epithelial cells

a Confirmation of the downregulated expression of target proteins by RNAi analysis using western blotting. Images are representative of two independent experiments with similar results. **b** Scatter plots comparing the tether force of MCF10A cells treated with the indicated RNAi. n = 60 (si-Control), n = 40 (si-RHOA), n = 28 (si-ERM), and n = 25 (si-SLK + STK10) cells pooled from three independent experiments. Mean ± SD. See also Fig. 2a. **c** Western blot of endogenous phospho-myosin light chain (pS19MLC), MLC, E-cadherin, vimentin, and β-actin levels in the indicated cells. Images are representative of two independent experiments with similar results. **d** Trajectories of cell centroids of the indicated cells tracked for 6 h in 2D. n = 21 (si-Control), n = 22 (si-RHOA), n = 17 (si-ERM), and n = 16 (si-SLK + STK10) cells from three independent experiments (see also Supplementary Movie 2). Right, scatter plots comparing their aspect ratios of n = 81 (si-Control), n = 98 (si-RHOA), n = 81 (si-ERM), and n = 85 (si-SLK + STK10) cells pooled from three independent experiments. Mean ± SD. **e** Quantification of AU565 or MCF7 cells that invaded through Matrigel. n = 6 fields from two independent experiments. Mean ± SD. **f** Proliferation rates of MCF10A cells treated with the indicated RNAi. Data are from the mean ± SD of three independent experiments. **g** Time-lapse images of MCF10A cells treated with the indicated siRNA in 3D. Yellow arrowheads indicate elongated moving cells (see also Supplementary Movie 3). Experiments were repeated three times independently with similar results. Scale bar, 20 μm. Statistical comparison to the appropriate control was performed using two-tailed Mann–Whitney test (**b, d**) and two-tailed Student's *t*-test (**e**). ****$P < 0.0001$.

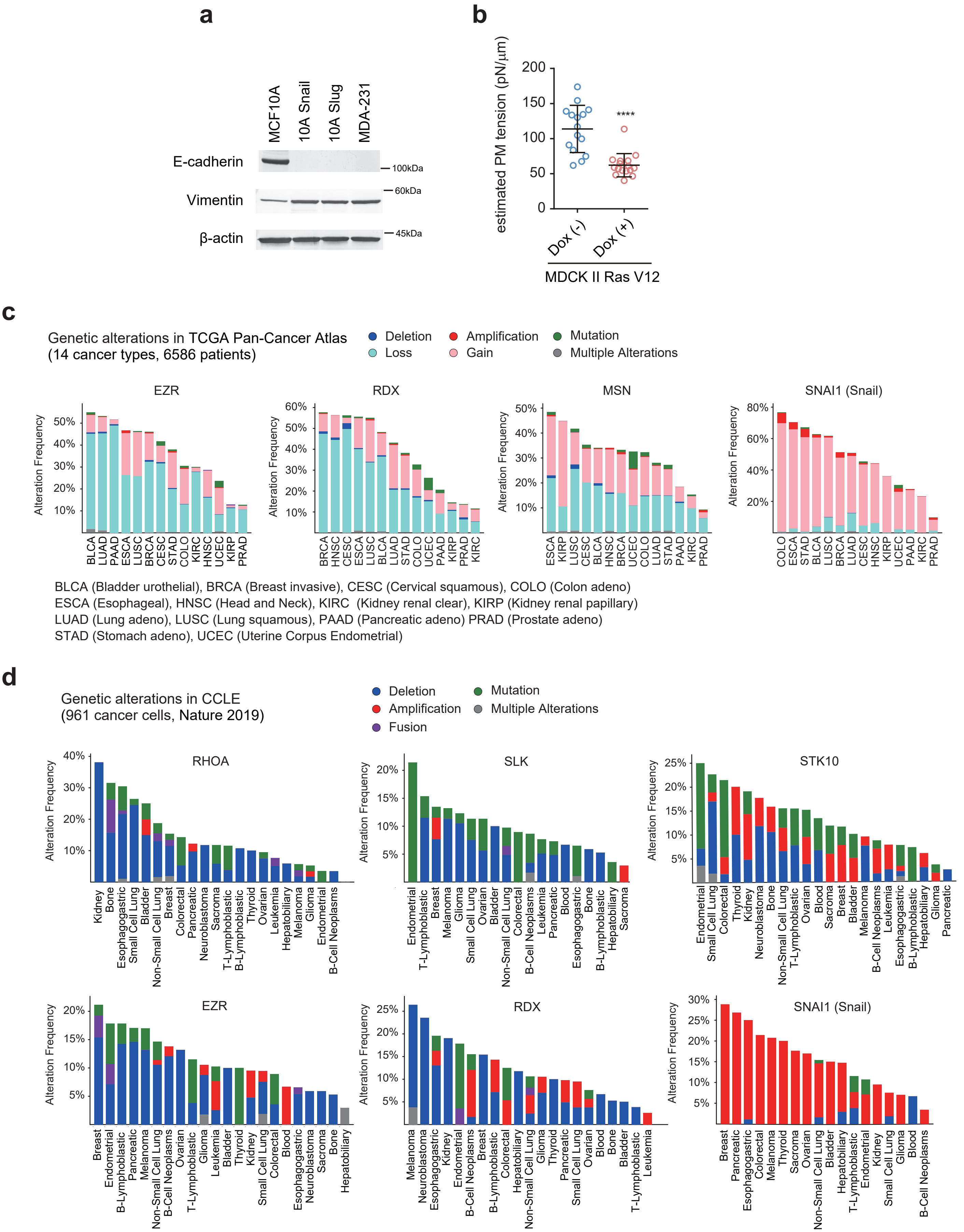

Tsujita et al, Supplementary Fig. 3

Supplementary Fig. 3 Downregulation of MCA regulators in cancer patients

a Western blot of endogenous E-cadherin, vimentin, and β-actin levels in the indicated cells. Images are representative of two independent experiments with similar results. **b** Scatter plots comparing the estimated PM tension of the indicated cells. n = 15 (MDCK II cells, Dox [-]) and n = 16 (MDCK II cells expressing RasV12, Dox [+]) cells pooled from three independent experiments. Mean ± SD. Two-tailed Mann–Whitney test. ****$P < 0.0001$. **c** Genetic alterations of the indicated genes across 14 human tumor types in TCGA data (6586 samples). **d** Genetic alterations of the indicated genes across 961 cancer cells in the Cancer Cell Line Encyclopedia (CCLE) data.

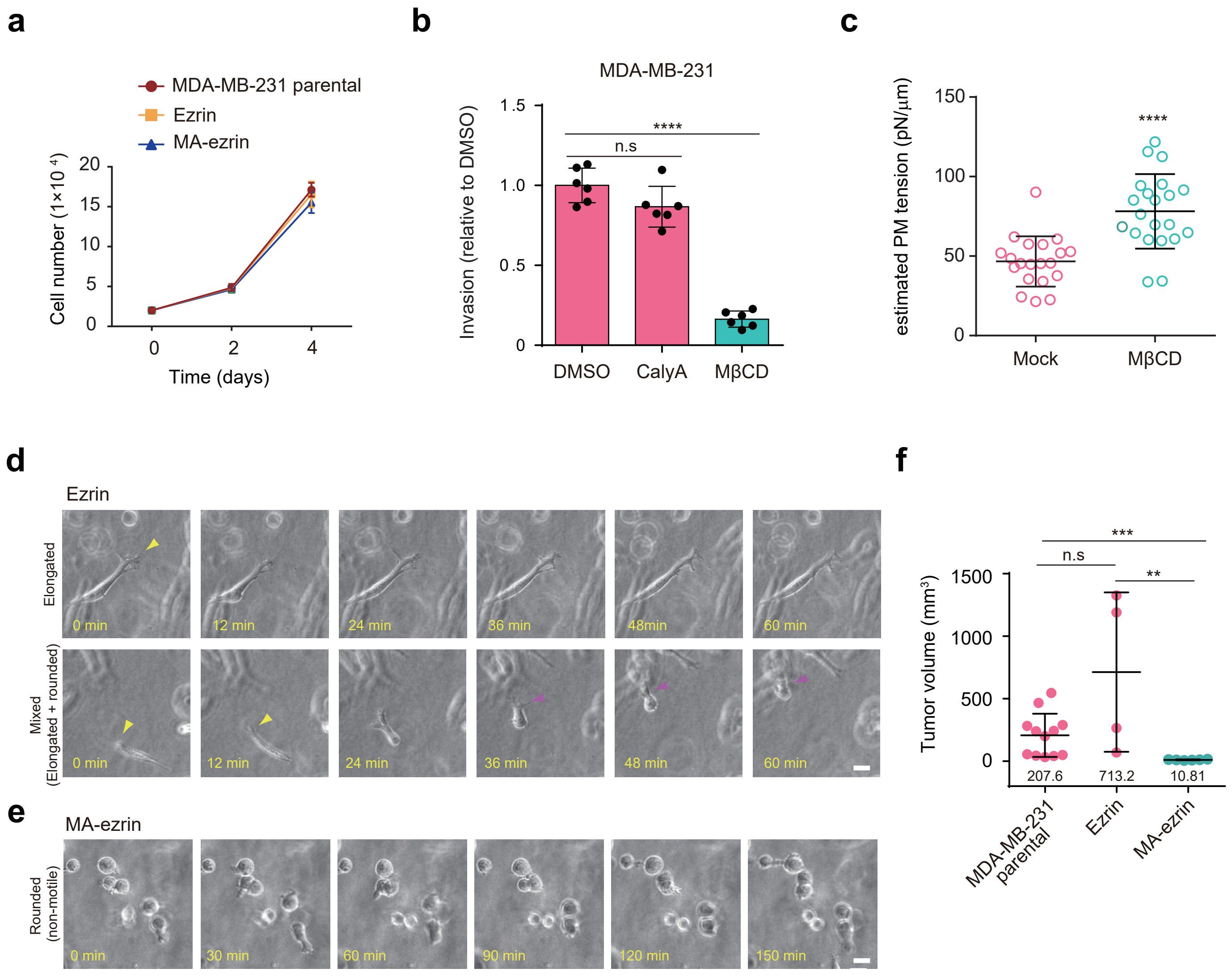

Tsujita et al, Supplementary Fig. 4

Supplementary Fig. 4 Increasing PM tension suppresses 3D migration

a Proliferation rates of MDA-MB-231 cells are expressed as indicated. Data are presented as the mean ± SD from three independent experiments. **b** Quantification of drug-treated cells that invaded through Matrigel. n = 6 fields from two independent experiments. Mean ± SD. n.s., not significant; ****P < 0.0001. **c** Scatter plots comparing the estimated PM tension of MDA-MB-231 cells treated with MβCD. n = 20 (Mock, water) and n = 21 (MβCD) cells pooled from three independent experiments. Mean ± SD. ****$P < 0.0001$. **d, e** Time-lapse images of MDA-MB-231 cells expressing ezrin (**d**) or MA-ezrin (**e**). In ezrin-expressing cells, yellow arrowheads indicate elongated moving cells, whereas magenta arrowheads indicate rounded moving cells. Experiments were repeated three times independently with similar results. Scale bars, 20 µm. **f** Primary tumor growth after injection of the indicated cells into the mammary fat pad. n = 12 mice (MDA-MB-231 parental), n =4 (ezrin) and n = 6 mice (MA-ezrin). Mean ± SD. The mean tumor volumes are shown in the graph. **$P = 0.0095$; ***$P = 0.0001$. Statistical analysis using two-tailed Student's *t*-test (**b, c**) and two-tailed Mann–Whitney test (**f**).

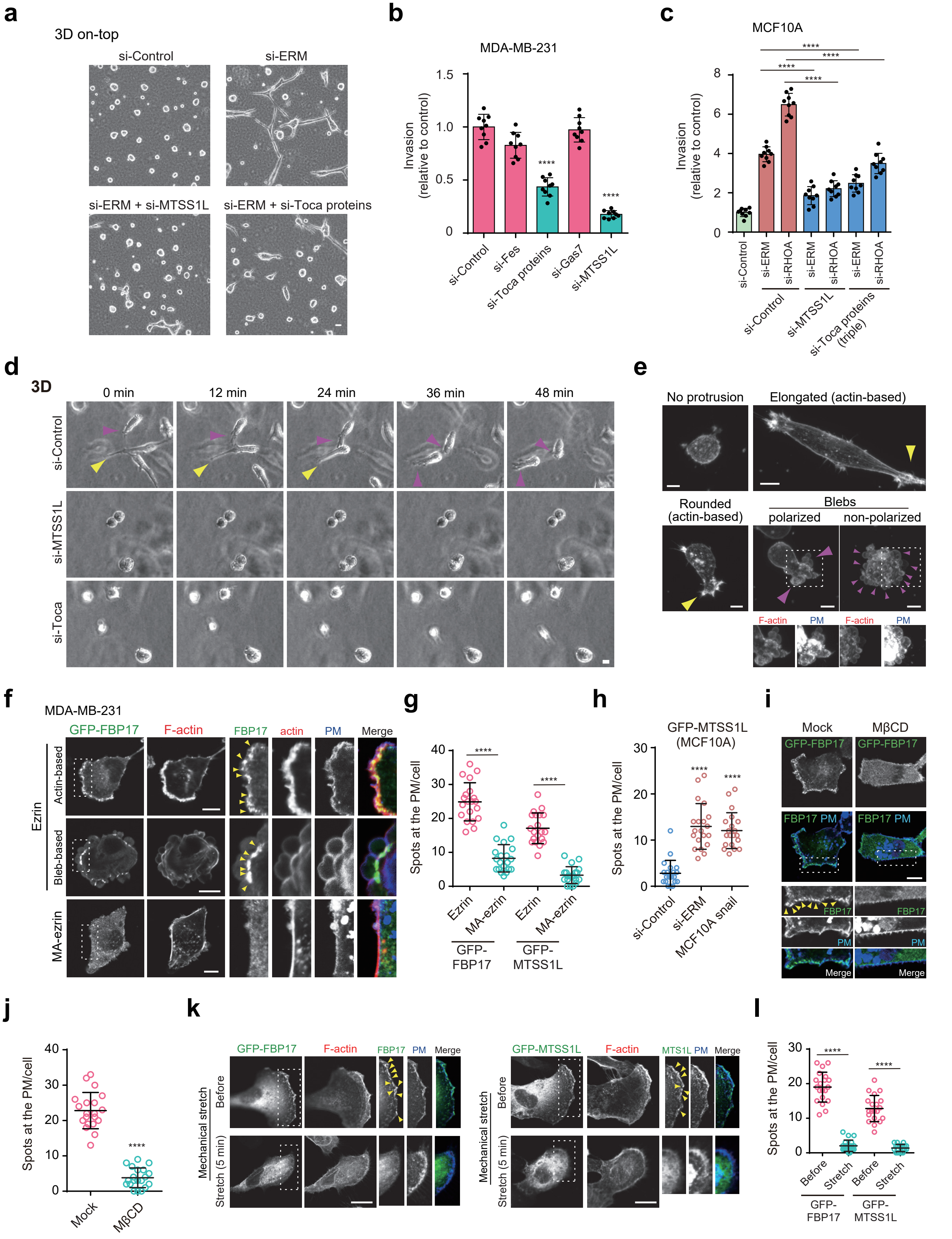
a
3D on-top
si-Control
si-ERM
si-ERM + si-MTSS1L
si-ERM + si-Toca proteins
b
MDA-MB-231
Invasion (relative to control)
si-Control
si-Fes
si-Toca proteins
si-Gas7
si-MTSS1L
c
MCF10A
Invasion (relative to control)
si-Control
si-ERM
si-RHOA
si-MTSS1L
si-Toca proteins (triple)
d
3D
0 min
12 min
24 min
36 min
48 min
si-Control
si-MTSS1L
si-Toca
e
No protrusion
Elongated (actin-based)
Rounded (actin-based)
Blebs
polarized
non-polarized
F-actin
PM
f
MDA-MB-231
GFP-FBP17
F-actin
FBP17
actin
PM
Merge
Ezrin
Actin-based
Bleb-based
MA-ezrin
g
Spots at the PM/cell
Ezrin
MA-ezrin
GFP-FBP17
GFP-MTSS1L
h
GFP-MTSS1L (MCF10A)
Spots at the PM/cell
si-Control
si-ERM
MCF10A snail
i
Mock
MβCD
GFP-FBP17
FBP17 PM
Merge
j
Spots at the PM/cell
Mock
MβCD
k
GFP-FBP17
F-actin
FBP17
PM
Merge
GFP-MTSS1L
MTS1L
Mechanical stretch
Before
Stretch (5 min)
l
Spots at the PM/cell
Before
Stretch
GFP-FBP17
GFP-MTSS1L

Supplementary Fig. 5. Homeostatic PM tension inhibits cancer cell migration by suppressing BAR protein assembly

a Phase-contrast images of MCF10A cells treated with the indicated RNAi grown in 3D on-top culture. Scale bar, 20 μm. **b, c** Quantification of the indicated RNAi-treated MDA-MB-231 cells (**b**) or MCF10A cells (**c**) that invaded through Matrigel. n = 9 fields from three independent experiments. Mean ± SD. **d** Time-lapse images of MDA-MB-231 cells treated with the indicated RNAi in 3D. Yellow arrowheads indicate elongated cells, whereas magenta arrowheads indicate rounded moving cells (see Supplementary Movie 5). Experiments were repeated three times independently with similar results. Scale bar, 20 μm**. e** Maximum intensity projections of control RNAi-treated MDA-MB-231 cells stained with phalloidin and WGA. Yellow and magenta arrowheads indicate actin- and bleb-based protrusions, respectively. Scale bars, 10 μm. These data are typical 3D images of protrusion phenotypes used in the quantification shown in Fig. 5e. Images are representative of three independent experiments. **f** Confocal images of ezrin or MA-ezrin cells expressing GFP-FBP17 stained with phalloidin and WGA. Yellow arrowheads indicate the FBP17 spots at the PM. Scale bars, 10 μm. **g** Quantification of GFP-FBP17 or GFP-MTSS1L puncta at the PM. n = 20 (GFP-FBP17) and n = 20 (GFP-MTSS1L) cells pooled from three independent experiments. **h** Quantification of GFP-MTSS1L puncta at the PM of n = 20 (si-Control), n = 20 (si-ERM), and n = 20 (Snail-expressing cells) cells pooled from three independent experiments. Mean± SD. **i** Confocal images of Mock (water)- or MβCD-treated MDA-MB-231 cells expressing GFP-FBP17 stained with WGA. Yellow arrowheads indicate the FBP17 spots at the PM. Scale bar, 10 μm. **j** Quantification of GFP-FBP17 puncta at the PM of n = 20 (Mock, water) and n = 20 (MβCD) cells pooled

from three independent experiments. Mean ± SD. **k** Confocal images of GFP-FBP17 (left) or GFP-MTSS1L (right) in MDA-MB-231 cells before and after mechanical stretching (20%). Yellow arrowheads indicate FBP17 or MTSS1L spots at the PM. Scale bars, 10 μm. **l** Quantification of (**k**). n = 20 (GFP-FBP17) or n = 20 (GFP-MTSS1L) cells pooled from two independent experiments. Mean ± SD. Statistical analysis using the two-tailed Student's *t*-test (**b, g, j**), one-way ANOVA with Tukey's multiple comparisons test (**c**), and two-tailed Mann–Whitney test (**h, l**). ****$P < 0.0001$.

Supplementary Table 1. List of primers for qPCR analysis in this study

Gene	Forward primer	Common reverse primer
Human-specific PTGER2	GCTGCTTCTCATTGTCTCGG	GCCAGGAGAATGAGGTGGTC
Mouse-specific PTGER2	CCTGCTGCTTATCGTGGCTG	GCCAGGAGAATGAGGTGGTC
