# Supplementary material for: Homeostatic membrane tension constrains cancer cell dissemination by counteracting BAR protein assembly
Source: Nat Commun. 2021 Oct 11;12:5930. doi: 10.1038/s41467-021-26156-4 (PMC8505629; doi:10.1038/s41467-021-26156-4)
Supplement: Supplementary file 3 — Description of Additional Supplementary Files [file 41467_2021_26156_MOESM3_ESM.pdf]

Description of Additional Supplementary Files

File Name: Supplementary Movie 1
Description: Membrane ruffling or blebbing cells under optical tweezers measurements. Time-lapse movies of MDA-MB-231 cells and Hs578T cells cultured on glass bottom dish during optical tweezers measurements. Arrows indicate membrane tether. The movies were taken at 1 frame per 2 seconds. Scale bars, 10 µm.

File Name: Supplementary Movie 2
Description: A decrease in PM tension induces epithelial cell migration in 2D. Time-lapse movies of MCF10A cells transfected with the indicated siRNA cultured on 2D glass substrates. The movies were taken at 1 frame per 5 min. Scale bar, 20 µm.

File Name: Supplementary Movie 3
Description: A decrease in PM tension induces epithelial cell migration in 3D environments. Time-lapse movies of MCF10A cells transfected with the indicated siRNA in a 3D collagen matrix. The movies were taken at 1 frame per 10 min. Scale bar, 20 µm.

File Name: Supplementary Movie 4
Description: Increasing PM tension is sufficient to suppress 3D migration of malignant cells. Time-lapse movies of ezrin, or MA-ezrin expressing MDA-MB231 cells in a 3D collagen matrix. The movies were taken at 1 frame per 6 min. Scale bar, 20 µm.

File Name: Supplementary Movie 5
Description: Depletion of BAR proteins suppresses 3D migration of malignant cells. Time-lapse movies of MDA-MB-231 cells transfected with the indicated siRNA in a 3D collagen matrix. The movies were taken at 1 frame per 6 min. Scale bar, 20 µm.

All movies were played at 10 frames per second.
