# Supplementary material for: Homeostatic membrane tension constrains cancer cell dissemination by counteracting BAR protein assembly
Source: Nat Commun. 2021 Oct 11;12:5930. doi: 10.1038/s41467-021-26156-4 (PMC8505629; doi:10.1038/s41467-021-26156-4)
Supplement: Supplementary file 9 — Reporting Summary [file 41467_2021_26156_MOESM9_ESM.pdf]

nature research

Corresponding author(s): Kazuya Tsujita

Last updated by author(s): Sep 13, 2021

Reporting Summary

Nature Research wishes to improve the reproducibility of the work that we publish. This form provides structure for consistency and transparency in reporting. For further information on Nature Research policies, see our Editorial Policies and the Editorial Policy Checklist.

Statistics

For all statistical analyses, confirm that the following items are present in the figure legend, table legend, main text, or Methods section.

n/a	Confirmed	
☐	☒	The exact sample size (*n*) for each experimental group/condition, given as a discrete number and unit of measurement
☐	☒	A statement on whether measurements were taken from distinct samples or whether the same sample was measured repeatedly
☐	☒	The statistical test(s) used AND whether they are one- or two-sided<br>*Only common tests should be described solely by name; describe more complex techniques in the Methods section.*
☒	☐	A description of all covariates tested
☐	☒	A description of any assumptions or corrections, such as tests of normality and adjustment for multiple comparisons
☐	☒	A full description of the statistical parameters including central tendency (e.g. means) or other basic estimates (e.g. regression coefficient) AND variation (e.g. standard deviation) or associated estimates of uncertainty (e.g. confidence intervals)
☐	☒	For null hypothesis testing, the test statistic (e.g. *F*, *t*, *r*) with confidence intervals, effect sizes, degrees of freedom and *P* value noted<br>*Give P values as exact values whenever suitable.*
☒	☐	For Bayesian analysis, information on the choice of priors and Markov chain Monte Carlo settings
☒	☐	For hierarchical and complex designs, identification of the appropriate level for tests and full reporting of outcomes
☒	☐	Estimates of effect sizes (e.g. Cohen's *d*, Pearson's *r*), indicating how they were calculated

Our web collection on statistics for biologists contains articles on many of the points above.

Software and code

Policy information about availability of computer code

Data collection	NanoTracker control software (JPK, version 3.0), FV10-ASW (Olympus), BZ-X Viewer (Keyence)
Data analysis	JPK Data Processing (JPK, version 2.5), Image J (NIH, version 1.46r), Imaris 8.0.2(Bitplane), Chemotaxis and Migration Tool (Ibidi, version 2.0),Prism 6 (GraphPad), Microsoft Excel 2010.

For manuscripts utilizing custom algorithms or software that are central to the research but not yet described in published literature, software must be made available to editors and reviewers. We strongly encourage code deposition in a community repository (e.g. GitHub). See the Nature Research guidelines for submitting code & software for further information.

Data

Policy information about availability of data

All manuscripts must include a data availability statement. This statement should provide the following information, where applicable:
- Accession codes, unique identifiers, or web links for publicly available datasets
- A list of figures that have associated raw data
- A description of any restrictions on data availability

Source data are provided with this paper. TCGA and CCLE datasets are available from the cBioPortal (http://www.cbioportal.org/). Clinical data sets of cancer patients are available from KMplot (http://www.kmplot.com). All the other data are available within the article and its Supplementary Information

Field-specific reporting

Please select the one below that is the best fit for your research. If you are not sure, read the appropriate sections before making your selection.

☒ Life sciences ☐ Behavioural & social sciences ☐ Ecological, evolutionary & environmental sciences

For a reference copy of the document with all sections, see nature.com/documents/nr-reporting-summary-flat.pdf

Life sciences study design

All studies must disclose on these points even when the disclosure is negative.

Sample size	No statistical method was used to define sample size. Sample size was chosen based on previous studies in the field (Lieber et al, Curr Biol 2013; Thottacherry et al, Nat Commun 2018; Serres et al, Dev Cell 2020; Ilina et al, Nat Cell Biol 2020). Sample size for each experiment is indicated in all figure legends.
Data exclusions	No data were excluded from analysis.
Replication	All experiments were repeated at three times independently, except for Fig. 5a, and Supplementary Figs. 1h; 2a,c, e; 3a; 4b; 5l (two independent experiments). All attempts at replication were successful.
Randomization	Methods of randomization were not used. For animal studies there were no covariates as all mice were of the same age and sex and were kept together in the same conditions. For in vitro experiments, cells were cultured under the same conditions and randomly selected.
Blinding	Same researcher performed the experiments and analyzed data, therefore, it was not blinding.

Reporting for specific materials, systems and methods

We require information from authors about some types of materials, experimental systems and methods used in many studies. Here, indicate whether each material, system or method listed is relevant to your study. If you are not sure if a list item applies to your research, read the appropriate section before selecting a response.

Materials & experimental systems

n/a	Involved in the study	
☐	☒	Antibodies
☐	☒	Eukaryotic cell lines
☒	☐	Palaeontology and archaeology
☐	☒	Animals and other organisms
☒	☐	Human research participants
☒	☐	Clinical data
☒	☐	Dual use research of concern

Methods

n/a	Involved in the study	
☒	☐	ChIP-seq
☒	☐	Flow cytometry
☒	☐	MRI-based neuroimaging

Antibodies

Antibodies used	The following antibodies were used: anti-ERM (rabbit polyclonal, 1:1000 for immunoblotting; #3142, Cell Signaling Technology [CST]); anti-phospho-ERM (rabbit monoclonal [48G2], 1:100 for immunostaining; #3726, CST); anti-RHOA (mouse monoclonal [26C4], 1:1000 for immunoblotting; #sc-418, Santa Cruz Biotechnology); anti-pS19 MLC (Phospho-myosin light chain 2 [Ser19]) (mouse monoclonal, 1:1000 for immunoblotting; #3675, CST); anti-MLC (rabbit polyclonal, 1:1000 for immunoblotting; #3672, CST); anti-E-cadherin (rabbit monoclonal [24E10], 1:1000 for immunoblotting; #3915, CST); anti-vimentin (rabbit monoclonal[D21H3], 1:1000 for immunoblotting; #5741, CST); anti-MTSS1L (rabbit polyclonal, 1:200 for immunoblotting; #NBP2-57037, Novus Biologicals); anti-FBP17 (FNBP1) (rabbit polyclonal, 1:1000 for immunoblotting, established in Dr P. De Camili Lab, Yale University); anti-CIP4 (TRIP10) (mouse monoclonal, 1:1000 for immunoblotting; #612556, BD Transduction Laboratories); anti-HA-Tag (rabbit monoclonal [C29F4], 1:100 for immunostaining; #3724, CST); anti-β-actin (rabbit polyclonal 1:2000 for immunoblotting; #PM053, MBL); goat anti-rabbit secondary antibody Alexa-Fluor-488-conjugated (#A11034, 1:500 for immunoblotting, Thermo Scientific), and goat anti-mouse secondary antibody Alexa-Fluor-488-conjugated (#A11029, 1:500 for immunoblotting, Thermo Scientific).
Validation	Western blotting<br>anti-ERM (#3142; species reactivity, Human) was validated by 67 refs as reported by the manufacturer (https://www.cellsignal.jp/products/primary-antibodies/ezrin-radixin-moesin-antibody/3142).<br>anti-RHOA (#sc-481; species reactivity, Human) was validated by over 1000 refs as reported by the manufacturer (https://www.scbt.com/p/rho-a-antibody-26c4?productCanUrl=rho-a-antibody-26c4&_requestid=3898493).<br>anti-pS19MLC (#3675; species reactivity, Human) was validated by over 200 refs as reported by the manufacturer (https://www.cellsignal.jp/products/primary-antibodies/phospho-myosin-light-chain-2-ser19-mouse-mab/3675).<br>anti-MLC (#3672; species reactivity, Human) was validated by over 200 refs as reported by the manufacturer (https://

www.cellsignal.jp/products/primary-antibodies/myosin-light-chain-2-antibody/3672).
anti-E-cadherin (#3915; species reactivity, Human) was validated by over 1500 refs as reported by the manufacturer (https://www.cellsignal.jp/products/primary-antibodies/e-cadherin-24e10-rabbit-mab/3195).
anti-vimentin (#5741; species reactivity, Human) was validated by over 1200 refs as reported by the manufacturer (https://www.cellsignal.jp/products/primary-antibodies/vimentin-d21h3-xp-rabbit-mab/5741).
anti-MTSS1L (NBP2-57037; species reactivity, Human) was validated by knockdown in this study.
anti-FBP17 (species reactivity, Human) was previously validated (Itoh et al, Dev Cell 2005).
anti-CIP4 (#612556; species reactivity, Human) was previously validated (Echarri et al, Nat Commun 2019).
anti-β-actin (#PM053; species reactivity, Human) was previously validated (Takahashi et al, Nat Commun 2015).
Immunofluorescence
anti-phospho-ERM (#3726; species reactivity, Human) was validated by 34 refs as reported by the manufacturer (https://www.cellsignal.jp/products/primary-antibodies/phospho-ezrin-thr567-radixin-thr564-moesin-thr558-48g2-rabbit-mab/3726).
anti-HA-tag (#3724) was validated by over 1000 refs as reported by the manufacturer (https://www.cellsignal.jp/products/primary-antibodies/ha-tag-c29f4-rabbit-mab/3724).

Eukaryotic cell lines

Policy information about cell lines

Cell line source(s)	MCF10A, MDA-MB-231, Hs578T, MCF7, AU565, PANC-1 were obtained from ATCC. PC-3 and IAR-2were from JCRB. MDCK II cells were previously described (Furuse et al, J Cell Biol 2001; gift from Dr M. Murata [University of Tokyo]). MDCK II cells carrying doxycycline-inducible RasV12 were previously described (Kon et al, Nat Cell Biol, 2017).
Authentication	Cell lines were not authenticated.
Mycoplasma contamination	All cell lines were tested for mycoplasma contamination and found negative.
Commonly misidentified lines (See ICLAC register)	No commonly misidentified cell lines were used.

Animals and other organisms

Policy information about studies involving animals; ARRIVE guidelines recommended for reporting animal research

Laboratory animals	6-week-old female BALB/c nu/nu mice were used. Mice were maintained in a temperature (23±1°C, 55±5% humidity) with a 12/12 hour light/dark cycle.
Wild animals	No wild animals were used in the study.
Field-collected samples	No field collected samples were used in the study.
Ethics oversight	All animal experiments were reviewed by the Institutional Ethics Committee and performed in compliance with the Guidelines for Laboratory Animal Research of the Tokyo University of Pharmacy and Life Sciences (Tokyo, Japan).

Note that full information on the approval of the study protocol must also be provided in the manuscript.
